# Assessing plastic ingestion in the White stork (*Ciconia ciconia*) through regurgitated pellets

**DOI:** 10.1007/s11356-025-35956-w

**Published:** 2025-01-29

**Authors:** Elena Ramos-Elvira, Alejandro López-García, Laura Osorio, Irene Colino-Freire, Rosa María Garcinuño-Martínez, Pilar Fernández-Hernando, José I. Aguirre

**Affiliations:** 1 Facultad de Ciencias Biológicas, Universidad Complutense de Madrid, Madrid, Spain; 2 Universidad Nacional a Distancia, Madrid, Spain

**Keywords:** Environmental pollution, Human impacts, Microplastics, Landfill, Terrestrial environments, Trophic efficiency

## Abstract

Pollution is one of the main factors that threaten biodiversity nowadays. Plastic waste is a global problem which impacts not only on the marine environment but also on the terrestrial one. Great amounts of this kind of refuse are compiled in landfills, where lots of avian species feed. In contrast to seabirds research, there are limited studies that have considered how plastic is being ingested by land birds even when they are being affected both physically and at an endocrine level. We tried to assess the number of plastics and microplastics ingested by individuals of a White stork (*Ciconia ciconia*) colony in central Spain by collecting regurgitated pellets. The chemical composition of the elements was determined, as well as the relation between the amount of ingested plastic by individuals and their use of a landfill. Our results show that 3.44% of the pellet was formed by plastic (*n* = 50). Polyethylene, polystyrene, polypropylene and PET were the most abundant polymers, all of them being potentially problematic to the organism according to the literature. Each polymer was identified by Fourier transform infrared attenuated total reflectance spectroscopy (FTIR-ATR). We observed that the total amount of ingested plastics was stable along the use of the landfill, meaning White storks obtained plastic not only from anthropogenic sources but also from natural areas, indicating its high rate of pollution. Our study remarks the importance of addressing plastic ingestion in White storks as well as other terrestrial species, not only to understand the possible damage to the population but also to the whole ecosystem.

## Introduction

The global impact of human activities on ecosystems is a defining feature of the Anthropocene (Lewis and Maslin 2015). Currently, several factors threaten biodiversity such as climate change or global pollution. Plastic, among various synthetic products, stands out as a major contributor to profoundly affecting the environment. Some negative consequences of this contaminant are due to their slow or near non-existent degradation, depending on their composition (Chamas et al. 2020). Furthermore, existing recycling methods lack the efficacy to remove these materials in the quantities they are being produced. It was estimated that approximately 1200 million tons of plastic will be present in the environment by 2050 (Welden 2020).

Plastic has an impact on various animal species in diverse ways: (a) new colonisations, as sessile fauna could travel across the ocean on plastic waste (Barnes and Milner 2005); (b) wounds, entanglements, or movement limitations that hinder the proper progression of the individual’s life (Jepsen and de Bruyn 2019); or (c) ingestion by similarities between plastics and real food (Santos et al. 2021). Not only are there physical aspects that affect the individuals but also chemical and hormonal ones, such as hepatic stress, impaired development, or bioaccumulation (Alabi et al. 2019). Even though there is a well-documented problematic associated to plastics, as seen in the introduction and discussion of this text, some authors state that plastic ingestion might not be as harmful as commonly claimed (Roman et al. 2019b).

Plastics are synthetic organic polymers which are mainly manufactured from chemical derivatives of petroleum. There are several types of plastic polymers, which can even be mixed to synthesize new materials. Most used ones are high-density polyethylene (HDPE), low-density polyethylene (LDPE), polypropylene (PP), polystyrene (PS), polyvinyl chloride (PVC) and polyethylene terephthalate (PET). Previous research shows that many of these polymers might affect animals and their body conditions by causing endocrine disruption, growth alteration, or even mortality (Lithner et al. 2012; Rochman et al. 2014; Au et al. 2015; Horn et al. 2020; Estrela et al. 2021). In addition to the compounds used in the manufacture of plastic polymers (monomer, crosslinker, etc.), they may contain derivatives and additives, known as plasticizers, which are chemical substances added to plastics to improve characteristics such as lowering the processing temperature, improving ductility, or to act as lubricants (Bialecka-Florjaczyka and Florjanczyk, 2007). An example is organophosphorus ester (OPE) which can be easily found in the environment, and it is identified as carcinogenic or neurotoxic (Wang et al. 2020). Other compounds used as additives are phthalates and bisphenols, which are endocrine disruptors (Braver-Sewradj et al. 2020; Wang and Qian 2021). These plasticizers can be released in nature while they are synthesized, processed, used and displaced (Bialecka-Florjaczyka and Florjanczyk 2007).

Microplastics are small fragments with a smaller size than 5 mm (Filella 2015). They are the result of plastic degradation, as it usually breaks down once it reaches the environment. Other fragments are intentionally synthesized for particular applications and are discarded directly into the environment, as waste from the cosmetics or pharmaceuticals industry, or from some other type of sector (Evode et al. 2021).

Previous research about plastic pollution and its impact on fauna is predominantly focused on the marine environment, e.g. Reusch (2014) and Peng et al. (2020). To date, studies on land are scarce; there is a considerable knowledge gap which needs to be closed (Jagiello et al. 2019; Santos et al. 2021). The effects on terrestrial fauna cannot be ignored and should be addressed since large quantities of plastics, microplastics and their derivatives end up on the soil (Bläsing and Amelung 2018). To broaden the understanding of this problem, it is crucial to explore the implications of plastic pollution on terrestrial fauna, as Nessi et al. did in 2022, when they studied pollution in Barn owl pellets at three different sites. It might be even more necessary when some generalist bird species have adapted to close contact with humans by taking benefit from some opportunities such as feeding from organic waste, which makes them especially sensitive to plastic pollution and so they could be valuable bioindicators to estimate environmental pollution (Furness and Camphuysen, 1997; Burger and Gochfeld 2004; Seress and Liker, 2015; Katlam et al., 2018).The White stork (*Ciconia ciconia*) is a partially migratory bird which had an increase on its Spanish populations due to the use of landfills as a feeding source and conservation projects (Tortosa et al. 2002; Martí, 2003; Molina and del Moral, 2005). Landfills provide nourishment in an abundant and predictable way for many bird species (López-García and Aguirre 2023). Nevertheless, landfill usage might result in the ingestion of plastics, potentially interfering with the animal system.

Regurgitated pellets are indigestible residues of food formed in the bird’s gizzard that many species eliminate some hours after the ingestion. They are composed of bones, insects’ exoskeletons and other organic and inorganic materials (Winkler et al. 2020). Thanks to the analysis of their chemical composition, the presence of plastics and their types of polymers, as well as other parameters such as the amount of mineral or organic matter that conform the pellet, can be verified. Pellets could serve as a valuable sampling method of immediate food intake of a particular individual. Clearly, gaining access to the entire digestive system might provide a more precise insight of the plastic consumption (Provencher et al. 2019). However, given the paramount importance of prioritizing animal welfare, non-invasive testing is essential; this way the individual can carry on their vital activities with no damage. So, collecting regurgitated pellets is an interesting choice for living White storks in order to not make any harm to any individual (Mikula et al 2023; Bjedov et al. 2024).

The aim of the present study is to assess the presence and identification of plastics and microplastics in regurgitated pellets from a bioindicator species, such as the White stork, and to relate it to the landfill usage of each individual. An attempt will also be made to assess whether the size and composition of the pellets are influenced by the dietary preferences of particular individuals.

Considering the high densities of plastic debris in landfills (Zhou et al. 2014), we predict that the collected samples will contain fragments of plastic and other anthropogenic materials. Similarly, we expect that individuals with more frequent use of the landfill will produce pellets with a higher plastic content.

## Materials and methods

### Study area

The study area was established in a White stork colony located in Soto del Real (40.74° N, 3.89° W), province of Madrid, Spain, 12 km away from a landfill (Colmenar Viejo, 40.66° N, 3.72° W), where White storks regularly feed. Since 1999, stork fledglings at the colony have been marked with PVC rings. In addition, weekly visits to the nearby landfill have been performed allowing identifying feeding individuals by their rings inside of it. This way, it is possible to establish a landfill use index (LUI) for each individual as a coefficient between the number of sightings of one particular bird and the number of total visits from a specific period of time (López-García et al. 2021).

To identify breeding individuals at each nest of the colony by their PVC rings, camera traps (Browning Patriot 24Mpx) were used. In total, 50 adults’ regurgitated pellet samples were collected at the nests and had their composition analysed. As in some pairs there was only one PVC-ringed member and since this species shows assortative mating behaviour (Barbraud and Barbraud 1999; Jagiello et al. 2018), we assumed both members of the breeding pair would make similar exploitation of the feeding habitat and, therefore, will have a similar LUI.

### Procedure for sample analysis

Pellet samples (*n* = 50) were collected in the surface of the nest during the period prior to egg hatching to ensure they belonged solely to breeding individuals. Samples were immediately frozen at − 18 °C until analysis. Once defrosted, they were dried at 50 °C for 24 h before processing, following the protocol by Provencher et al. (2019). After drying, they were weighed and measured to obtain a volume value (height × width × length in mm^3^). Then, we manually disaggregated them with metal tweezers. Any large plastics and identifiable organic and inorganic materials such as arthropod exoskeletons, bones, glass, or pebbles were separated. These larger plastics were stored in Petri dishes for further analysis and identification.

To isolate possible smaller plastics and microplastics masked by organic matter, a digestion study was carried out in different media (basic, acidic and oxidizing) capable of destroying organic matter without damaging plastic material. In order to do this, the samples—already free of large particles—underwent different digestions, hot and with constant orbital agitation, using the following attacking mixtures described in the literature: (a) Fenton’s reagent (0.05 M FeSO_4_ and H_2_O_2_); (b) 20% potassium hydroxide (KOH) and 30% hydrogen peroxide (H_2_O_2_); and (c) 30% KOH, 30% H_2_O_2_ (Prata et al., 2019; Provencher et al. 2019).

Considering the varied composition of the samples under study, where some contained a substantial amount of vegetal and animal material while others did not, none of the attacking reactive mixtures studied were clearly effective in the total removal of organic matter for all the samples studied. Subsequently, a 30% nitric acid (HNO_3_) solution was used, which markedly improved digestion although it remained insufficient. Continuing with the optimization, the best digestion was based in the use of acid and oxidant mixture at the same time: 20% HNO_3_ and 30% H_2_O_2_ as the attacking reagent, with heating at 45 °C and with orbital shaking at 120 rpm for 15 h. This digestion allowed eliminating the organic matter without causing damage to the plastics and thus the possible plastic materials present in the samples were able to be isolated.

To ensure that this process did not interfere with the plastic composition, we carried out a control digestion under identical conditions of various polymers such as polyvinyl chloride (PVC), polypropylene (PP), high-density polyethylene (HDPE), low-density polyethylene (LDPE) and polystyrene (PS). Two samples per polymer were weighed before and after the digestion process; the weight remained constant. In addition, FTIR-ATR spectra of the polymers were carried out before and after digestion and no spectral differences were observed, which confirmed that the polymers were not affected by the attacking reagent.

The pellet samples, already digested, were filtered on filter paper with a Büchner funnel and a Kitasato flask. The retained material on the filter was dried and the plastic materials were extracted and deposited in a Petri dish. Prior to FTIR-ATR analysis, the extracted plastics and microplastics were cleaned with methanol (CH_3_OH) in an ultrasonic bath for 20 min.

Visual identification of plastics and microplastics was carried out using a stereoscopic microscope (Motic SMZ-171) with a camera (Moticam S6) to enhance visualization and facilitate measurements and photographs of the plastic particles. The height and width of each plastic were measured to obtain a total area value (total sum of height × width in mm^2^) for each pellet sample.

To identify the types of polymers that make up the plastics and microplastics found in the samples, Fourier transform infrared spectroscopy with attenuated total reflectance (FTIR-ATR) (Jasco FT/IR-4100) was used. The infrared spectra obtained from the possible plastic and microplastic particles were compared with a library of reference spectra, created by own measurements in the FTIR-ATR using standards.

### Statistical analysis

To characterize pellet size, we constructed a regression model with weight and volume of the regurgitated pellet. Another regression model was used to study the volume of the pellet and its relation to the ingested organic matter. In order to assess if the use of the landfill or the individual’s age had an influence in the pellet’s weight, a generalized linear model (GLM) (Gaussian distribution) was used. To further analyse the pellet’s content and its relation to the individual and its LUI, another GLM was performed using the organic matter percentage controlled by the age and the pellet’s volume. This organic matter percentage is a measure of naturality since it involves the weight of bones, twigs, exoskeletons, shells, etc. found in the regurgitated pellets. To establish a relation between the ingested plastic and the use of the landfill, a GLM was also executed. R and RStudio (version 4.2.1) were used to perform all these analyses.

## Results

### Regurgitated pellet characteristics

A total of 50 pellets were measured and weighed after drying, resulting in the data of Table 1. It was observed that larger pellets exhibited greater weights (*F* = 29.72; *p* < 0.0001). Larger pellets contained higher quantities of organic matter (*F* = 40.91; *p* < 0.0001).

**Table 1:** Pellet size and volume characteristics

	Weight (g)	Length (mm)	Height (mm)	Width (mm)	Volume (mm^3^)
Mean	12.32	43.79	31.23	24.60	34,132.17
Interval (max–min)	23.02–4.19	59.01–29.21	42.88–16.60	38.93–11.28	66,073.52–14,048.56

**Fig. 1:**
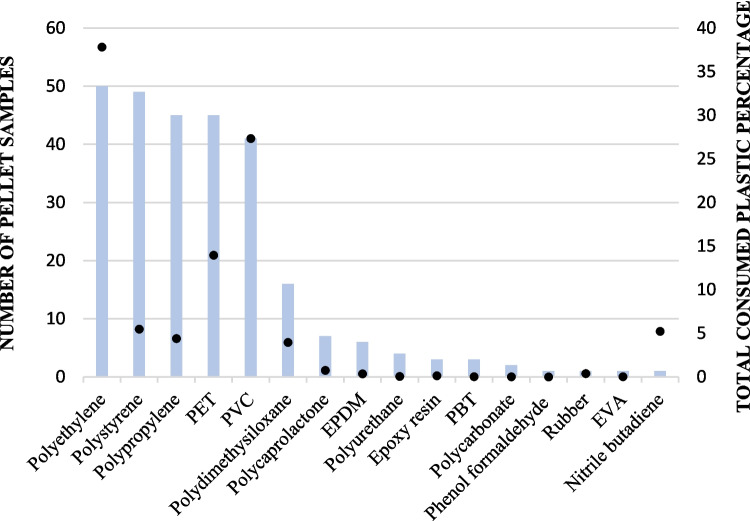
Plastic consumption by White storks. Number of pellet samples where the plastic polymer was present represented by solid bars and total polymer consumption percentage represented by solid dots. Sample size = 50

**Fig. 2:**
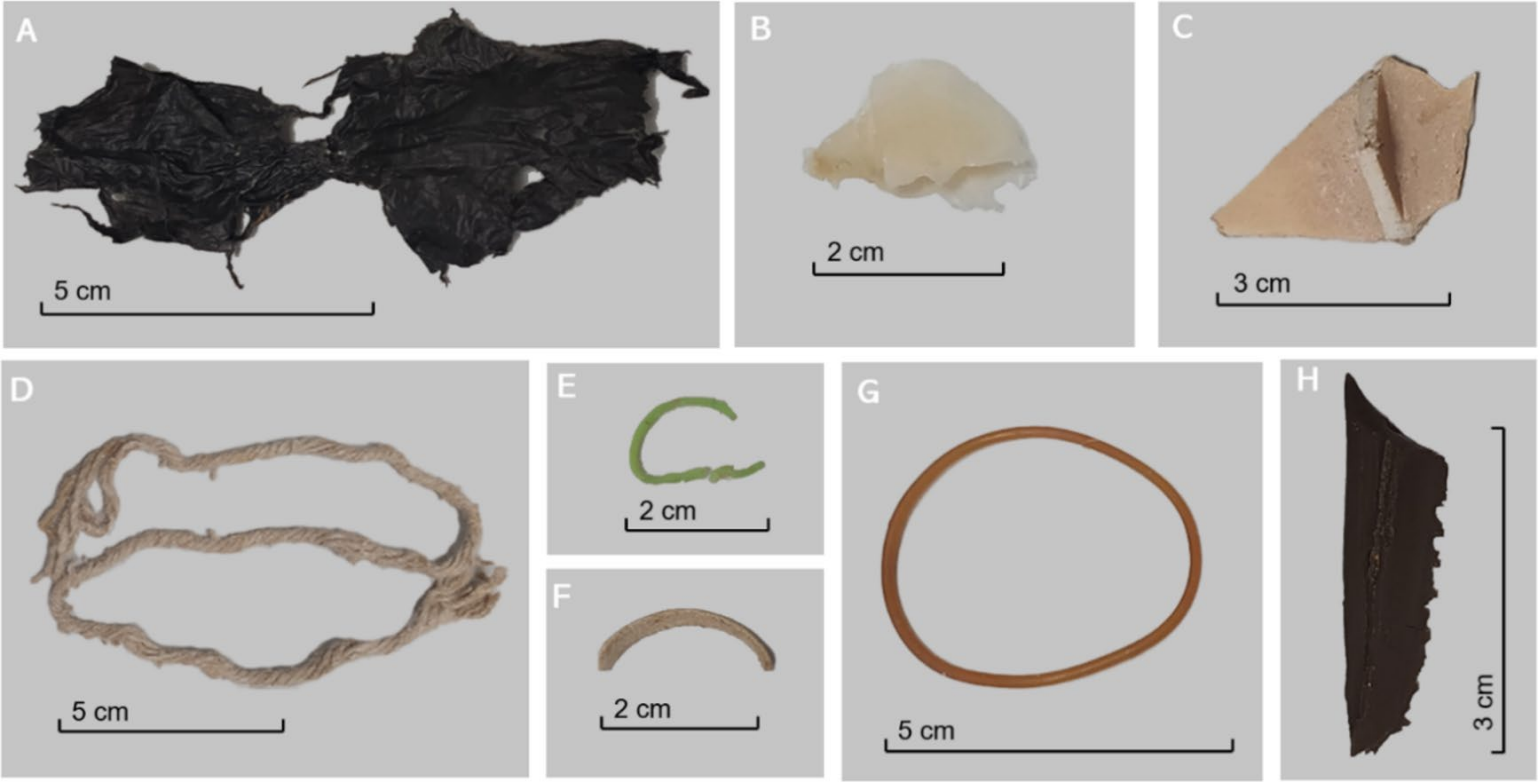
Plastic elements collected from regurgitated pellets before the digestion process. Scale in centimeters. **A** Polyethylene. **B** Polydimethylsiloxane. **C** Polypropylene. **D** PET. **E** Polystyrene. **F** Polyethylene. **G** Rubber. **H** Polydimethylsiloxane. Note that the same plastic polymer can show different presentations (**A** and **F**; **B** and **H**)

**Fig. 3:**
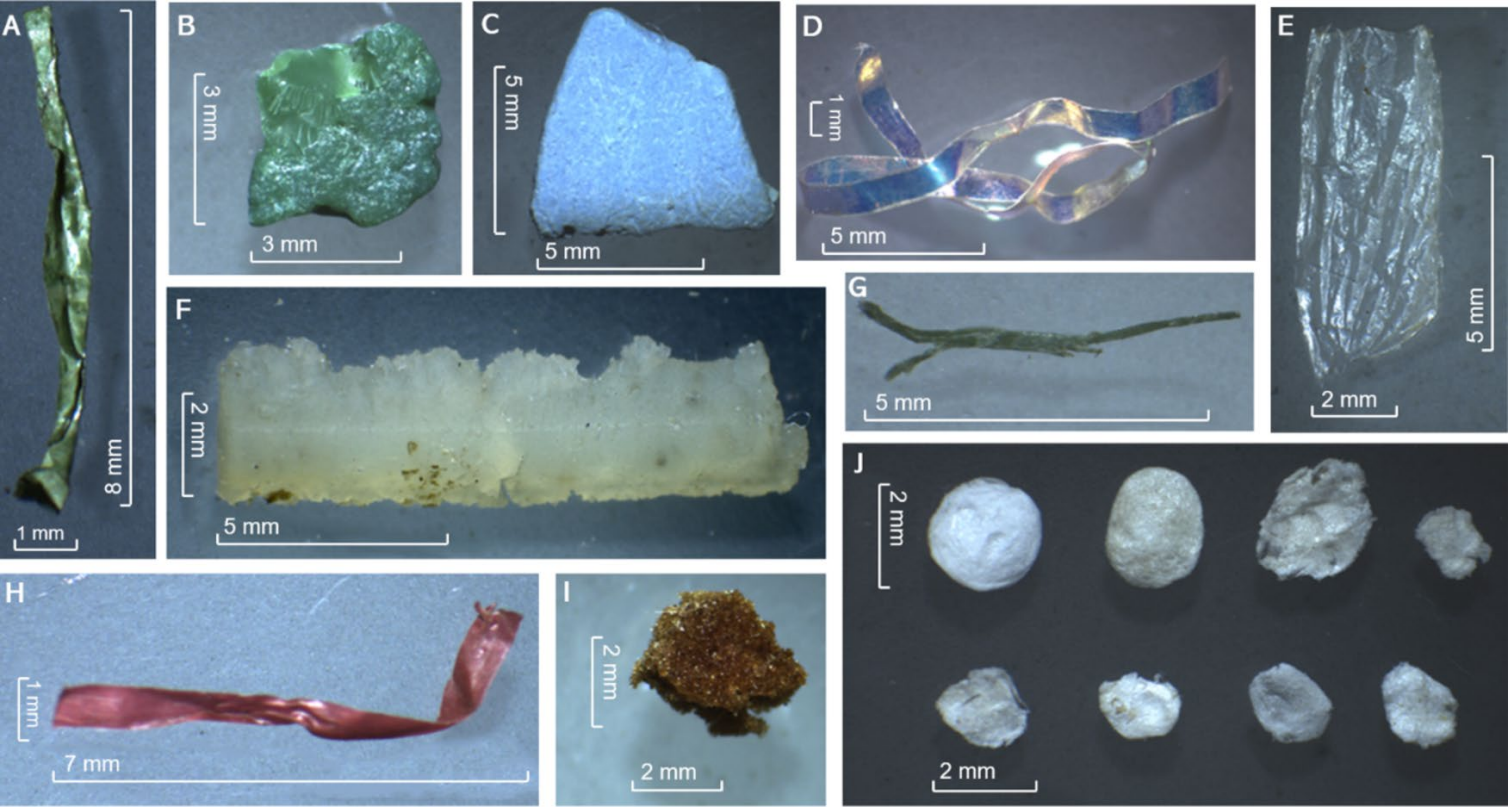
Plastic elements collected from regurgitated pellets after the digestion process. Scale in millimeters. **A** PVC. **B** Polyethylene. **C** PVC. **D** PET. **E** Polyethylene. **F** Polydimethylsiloxane. **G** Polyethylene. **H** Polyethylene. **I** Polyurethane. **J** Polystyrene. Note that the same plastic polymer can show different presentations (**A** and **C**, **B**, **E**, **G** and **H**)

In terms of pellet composition, the primary constituents of the regurgitated pellet were organic and mineral matter. Nonetheless, there was an average of 3.44% of plastic materials, as a percentage by weight (Table 2). Every single pellet analysed (n = 50) contained some plastic particles. The most common polymer was polyethylene, present in all 50 pellets, followed by polystyrene (*n* = 49), polypropylene and PET (*n* = 45) and PVC *(n* = 41). The polymers that constituted a larger proportion of the total plastic consumption were polyethylene (37.82%), PVC (27.35%) and PET (13.95%) (Fig. 1). A distinction between the presence of polymers in the pellet samples and the overall polymer consumption percentage is necessary due to variations in the sizes and rates of plastic elements (Fig. 2 and 3). For instance, pellet sample 14 contained part of a nitrile glove which increased excessively that polymer percentage (5.23%) in the complete sample size (no additional nitrile particles were detected in the remaining samples). Refer to Fig. 1 for graphical explanation.

**Table 2:** Pellet composition. Percentage of the organic matter fraction, the mineral fraction and plastic fraction

	Organic	Mineral	Plastic
Mean	50.12	46.43	3.44
Interval (max–min)	86.22–15.77	81.01–12.00	27.64–0.003

In total, 1680 pieces of plastic and microplastic were found, measured and analysed to determine their polymeric composition. Figures 2 and 3 show images of plastic elements collected.

### Statistical results

**Fig. 4:**
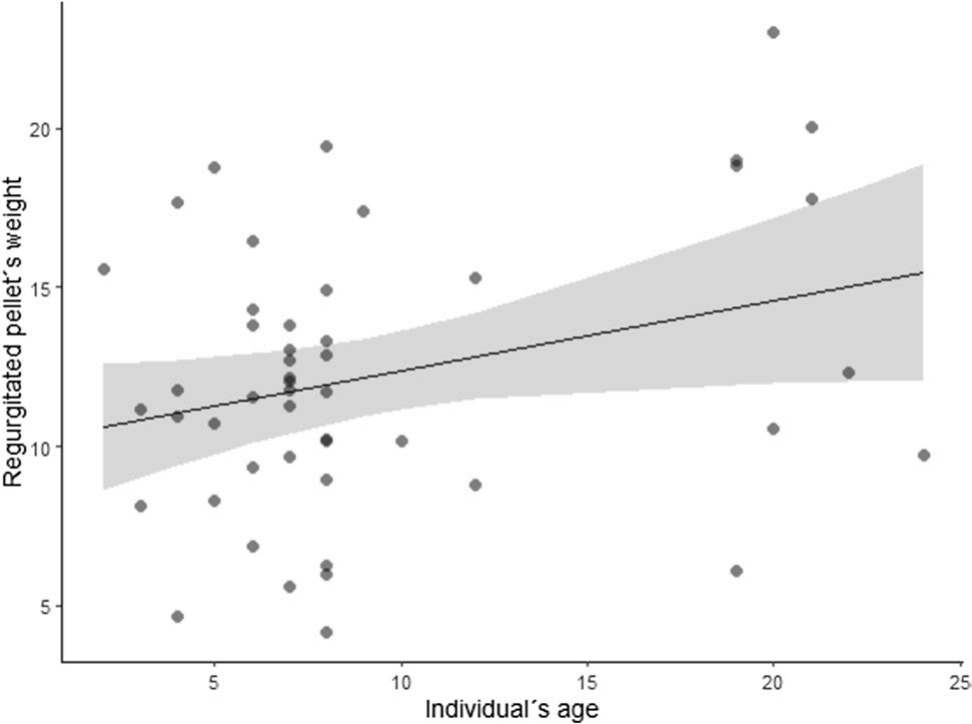
Graph that shows how older individuals produce larger pellets

**Fig. 5:**
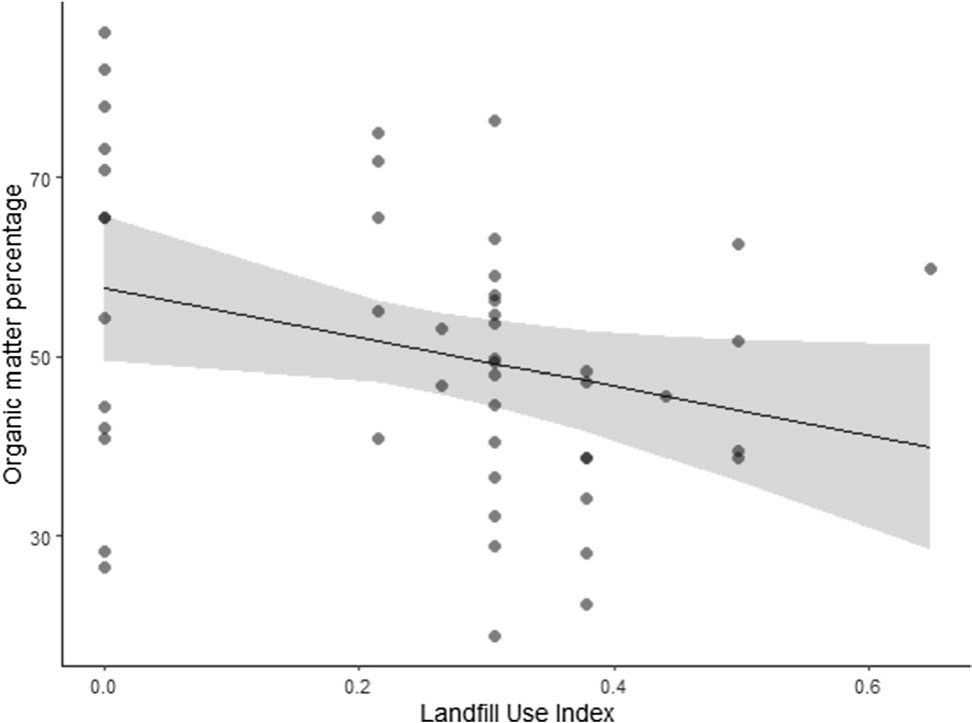
Graph that shows how the organic matter percentage decreases along the LUI

Significant differences in pellets size were found between the individual’s ages (*F* = 4.2213; *p* = 0.0456; Fig. 4) where older individuals tend to form bigger pellets. However, the LUI had no effect on the pellet’s weight (*F* = 0.0336; *p* = 0.8554). The organic matter percentage showed marginally significant differences (*F* = 3.7418; *p* = 0.0594; Fig. 5) for the LUI being controlled by the volume and age. The higher the organic percentage was, the lower the LUI.

There were no significant differences between the consumed plastic area percentage and the LUI (*F* = 0.0722; *p* = 0.7894). The plastic consumption seems to remain constant not only along the landfill use but also when there is no use (LUI = 0).

## Discussion

This research demonstrates that pellets can be a reliable and non-invasive tool to determine plastic presence in White storks’ diet. It enables the assessment of both the quantity of plastic materials consumed and their polymer composition. Moreover, they might serve as a proxy of plastic affection in the terrestrial environment.

Pellet samples’ weight increased with the volume of the sample, with results indicating that older individuals regurgitate larger and heavier pellets compared to younger ones. Larger pellets could serve as an indicator of extended foraging time periods and, potentially, a greater amount of food brought to the nest. This foraging may imply more time to spend defending the nest or incubating the eggs since individuals following this strategy would be reducing the foraging energy cost by reducing its frequency of leaving the nest. Storks usually have a higher food intake when there is greater food availability. Therefore, these results would follow an energy-maximizing strategy (Alonso et al. 1991; Zurell et al. 2015). Larger pellets might also indicate a higher ingestion of non-digestible organic elements, since the results show how larger pellets contained a greater proportion of organic material, such as bones, exoskeletons or shells. Nonetheless, it is noteworthy that the size of the pellet showed no relation to landfill usage. Thus, the size of the White stork pellets appears to be unaffected by the type of food individuals ingest and form them with.

Regarding pellet’s composition, the organic matter percentage had marginally significant differences following the LUI and being controlled by the volume of the pellet and the age of the individual. It might serve as a tool to discern the naturality of the feeding process. A higher percentage of organic matter indicates a greater presence of natural food components (involving exoskeletons, bones, shells and vegetation), such as White storks ingest as they typically forage (Johst et al. 2001; Chenchouni et al. 2015). Conversely, in anthropogenic food sources like landfills, the available food primarily consists in pre-processed human leftovers (e.g. meat or fish scraps) which are easily digestible, resulting in a lower organic matter percentage (Krook et al. 2012; López-García and Aguirre 2023). Therefore, organic matter percentage in pellets could be interpreted as a feeding indicator: lower levels in individuals might indicate foraging at a more efficient feeding source with less natural organic remains present in their pellets.

Despite considerable differences in the range of plastic percentages within the pellets (max. 27.64%; min. 0.003%), plastic material was detected in every single sample, mainly polyethylene, polystyrene, polypropylene and PET. Similarly, sea birds are generally related to polyethylene (this type of polymer usually being the most common) and polypropylene. Some others such as PVC or polystyrene are also noteworthy (Amélineau et al. 2016; Kühn et al. 2021; Teboul et al. 2021; Robuck et al. 2022). However, PET is usually not found, contrary to our results in White stork, a terrestrial species.

Furthermore, it should also be taken into consideration that plastics might serve as potential carriers for contaminants and chemicals due to their size and their absorption characteristics (Thompson et al. 2009), which can adversely affect wildlife by releasing these substances into the animals’ system. Regardless, the polymers identified in this research may induce biochemical changes in the individual’s organs leading to complications, as observed in various bird species as Japanese quails (*Coturnix japonica*) following polystyrene ingestion (de Souza et al. 2022), or in American black vultures (*Coragyps atratus*), exposed to polyethylene and polystyrene, where although body conditions remained constant biochemical alterations did occur (Cunha et al. 2022). While birds seem to not have been specifically tested for PET or PVC toxicity, studies on humans and other animals such as snails, fish or crustaceans have been reported to show alterations in their organism due to their consumption (Cormier et al. 2021; Dhaka et al. 2022). Therefore, plastic consumption in White storks may be causing some disruptions in their physiological conditions that are currently not fully understood.

As mentioned earlier, not only the chemical constituents of the plastic or the presence of contaminants pose a threat to the organism, but also the shape (sharp, splintered, etc.) and the hardness of the polymer can cause obstruction and other damage to the animal’s digestive system, potentially resulting in death. Such incidents have been reported in different avian species, including White storks, Northern gannet (*Morus bassanus*), several Shearwaters (*Procellaridae*) and Albatrosses (*Diomedeidae*) (Pierce et al., 2004; Hutton et al., 2008; Henry et al. 2011; Roman et al. 2019a, 2021).

Nonetheless, no significant differences were observed between the plastic elements found in the samples and the LUI, which might suggest that White storks not only feed from plastic on landfills but also on natural areas where there is not such an evident plastic source. It is well-documented that plastic debris in landfills is highly common (Zhou et al. 2014), potentially facilitating plastic consumption if individuals cannot easily distinguish between food and plastic items, as food wrapped in plastic films or if it has small plastic items stuck to it. For instance, research has shown that when White stork colonies are located at a greater distance from landfills than the typical foraging area, fewer plastic materials are found in the stomach of the individuals and also in their nests (Henry et al. 2011). As our findings indicate, landfills are not the only factor involved in plastic consumption. Litter in nature is a highly often occurrence which might lead to birds’ consumption. As previous studies have proven before, there is a relation between debris material in nests and human influence (Jagiello et al. 2019). Further research should be needed to assess and explain whether White storks are deliberately selecting plastics to eat or else they are ingesting them accidentally, although a combination of both behaviours might be the final answer since there have already been personal observations.

It is imperative to develop non-invasive methodologies that allow the research of plastics in avian species without resorting to post-mortem examination, as it is required when studying the whole digestive system. By using regurgitated pellets and keeping track of the presence of plastics or other materials as construction debris, metal elements, clothes, etc., it becomes feasible to assess the anthropogenic impact. This method not only enables to study the general effect on birds but also provides insights into the ecosystem and its wildlife, as the presence of these components in pellets imply an availability on the environment (Nessi et al. 2022; Cano-Povedano et al. 2023). These elements might have been gathered from synthetic sources such as landfills or from natural landscapes, indicating a high plastic pollution in the environment and it being in need of protective measures. It is an interesting starting point for future research as the impact of plastic pollution on terrestrial birds remains relatively understudied compared to the marine counterparts.

## Conclusion

This research highlights the importance of using regurgitated pellets as a method for studying plastic pollution. Synthetic polymers such as polyethylene, polystyrene or PET, which were the most abundant elements in the collected samples, have been reported to negatively impact birds through physical damage, organ alteration or biochemical changes.

In relation to landfill usage, plastic consumption appears to be consistent among individual birds. If White storks are ingesting plastic regardless of their frequency of visits to landfills, it suggests that many natural sources are highly polluted with plastics, up to the point where they easily feed on it. Alternatively, there may be a change in behaviour where White storks are intentionally ingesting plastics. Further research should be carried out to clarify this finding.

Nonetheless, the larger amount of organic matter percentage found in the regurgitated pellets relates to a reduced landfill usage, indicating a greater level of natural feeding behaviour.

It is paramount to approach plastic pollution and its effects on different species. Using White stork as a bioindicator species could allow policy-makers to improve local management measures and to take into consideration the conservation of the environment.

## Data Availability

All data available upon request.
