# Correction to: Assessing plastic ingestion in the White stork (*Ciconia ciconia*) through regurgitated pellets

**DOI:** 10.1007/s11356-025-36943-x

**Published:** 2025-11-01

**Authors:** Elena Ramos‑Elvira, Alejandro López‑García, Laura Osorio, Irene Colino‑Freire, Rosa María Garcinuño‑Martínez, Pilar Fernández‑Hernando, José I. Aguirre

**Affiliations:** 1 Facultad de Ciencias Biológicas, Universidad Complutense de Madrid, Madrid, Spain; 2 Universidad Nacional de Educación a Distancia, Madrid, Spain

**Correction to: Environmental Science and Pollution Research (2025) 32:4502–4510**

10.1007/s11356-025-35956-w

The article Assessing plastic ingestion in the White stork (Ciconia ciconia) through regurgitated pellets written by Elena Ramos‑Elvira, Alejandro López‑García, Laura Osorio, Irene Colino‑Freire, Rosa María Garcinuño‑Martínez, Pilar Fernández‑Hernando and José I. Aguirre was originally published under exclusive license to ©The Author(s), under exclusive licence to Springer-Verlag GmbH Germany, part of Springer Nature. As a result of the subsequent decision to publish the article under the open access model, the article’s copyright notice was changed on 27 August 2025 to ©The Author(s) 2025 and the article is now distributed under a Creative Commons Attribution 4.0 International License, which permits use, sharing, adaptation, distribution and reproduction in any medium or format, as long as you give appropriate credit to the original author(s) and the source, provide a link to the Creative Commons licence, and indicate if changes were made. The images or other third party material in this article are included in the article's Creative Commons licence, unless indicated otherwise in a credit line to the material. If material is not included in the article's Creative Commons licence and your intended use is not permitted by statutory regulation or exceeds the permitted use, you will need to obtain permission directly from the copyright holder. To view a copy of this licence, visit http://creativecommons.org/licenses/by/4.0/.

The Original article has been corrected.

